# Phenolic Compounds Impact on Rheumatoid Arthritis, Inflammatory Bowel Disease and Microbiota Modulation

**DOI:** 10.3390/pharmaceutics13020145

**Published:** 2021-01-22

**Authors:** Rosa Direito, João Rocha, Bruno Sepodes, Maria Eduardo-Figueira

**Affiliations:** 1 Research Institute for Medicines (iMed.ULisboa), Faculdade de Farmácia, Universidade de Lisboa, Av. Prof. Gama Pinto, 1649-003 Lisbon, Portugal; jrocha@ff.ulisboa.pt (J.R.); bsepodes@ff.ulisboa.pt (B.S.); efigueira@ff.ulisboa.pt (M.E.-F.); 2 Department of Pharmacy, Pharmacology and Health Technologies, Faculdade de Farmácia, Universidade de Lisboa, Av. Prof. Gama Pinto, 1649-003 Lisbon, Portugal; 3 Department of Pharmaceutical Sciences and Medicines, Faculdade de Farmácia, Universidade de Lisboa, Av. Prof. Gama Pinto, 1649-003 Lisbon, Portugal

**Keywords:** diet, inflammation, inflammatory bowel disease, colitis, rheumatoid arthritis, health systems, phenolic compounds, nutrition, microbiota, nanoencapsulation

## Abstract

Non-communicable chronic diseases (NCDs) are nowadays the principal cause of death, especially in most industrialized nations. These illnesses have increased exponentially with the consumption of diets very high in fat and sugar, not to mention stress and physical inactivity among other factors. The potential impact of suboptimal diets on NCDs’ morbidity and mortality rates brings to the forefront the necessity for a new way of improving dietary habits. The literature provides extensive scientific work that presents evidence that phenolic compounds from diets have antioxidant, anti-inflammatory and antiproliferative activities that impact human health. Gut microbiota modulation by some phenolic compounds leads to favorable changes in abundance, diversity, and in the immune system. However, polyphenol’s limited bioavailability needs to be overcome, highlighting their application in new delivery systems and providing their health benefits in well-established ways such as health maintenance, treatment or adjuvant to conventional pharmacological treatments. In this context, novel dietary approaches, including new food supplements, have emerged to prevent diseases and preserve health.

## 1. Introduction

Non-communicable diseases (NCDs) are chronic diseases that are non-infectious and non-transmissible, the most common of which are obesity, diabetes, cardiovascular, cancer, chronic respiratory and neurological diseases. Altogether, they are the most common causes of debility and death in the modern world, especially in the most industrialized countries. Oxidative stress, inflammation, and mitochondrial alterations are inextricably linked, performing a major part in the beginning and development of NCDs [1]. Due to this feature, they are most of the time called inflammatory chronic diseases, as is the case throughout this review. It is thus conceivable that nutritional or pharmacological manipulation of inflammation and oxidation permits for a significant decrease in the debility and mortality associated to these diseases [1].

These illnesses have increased exponentially as a result of the adherence to certain lifestyles, especially the consumption of diets very high in fat and/or sugar, stress, and physical inactivity, among other factors [2,3,4,5]. Between 2005 and 2015, the number of deaths from NCDs increased from 65% in 2005 to 71% in 2015 at a global level. Cancer was responsible for 14% of deaths in 2005, a number which in 2015 increased to 16% [6]. By 2015, there had been almost 18 million cancer cases around the world and nearly 9 million deaths, with cancer cases increasing by 33% between 2005 and 2015, and population aging contributing 16% [7]. Internationally, communities have responded to this health threat, with significant advances realized with the 2011 United Nations’ political declaration on NCD prevention and control [8], the World Health Organization’s Global Action Plan for the Prevention and Control of NCDs 2013–2020 [9] and the integration of NCDs in the Sustainable Development Goals for 2030 [10].

The Global Burden of Diseases, Injuries, and Risk Factors Study (GBD 2017 Diet Collaborators) assessed the ingesting of major nutrients and foods by almost 200 countries and measured the effects that their suboptimal consumption can have on NCD morbidity and mortality rates. Fifteen dietary characteristics were chosen for their impacts on disability and mortality from cancer, diabetes, and cardiovascular diseases across the 195 nations surveyed [5]. Adding to their previous findings [11], the GBD study brought to light important evidence-based dietary allegations and their impact on human health. The ingestion of a healthy diet was suboptimal worldwide (i.e., low consumption of nuts and seeds, whole grains, and milk), while the consumption of less healthy diets (i.e., sugary beverages, processed, red meat, and salt) was more common. Possibly accountable for this high burden of disease are the evaluated dietary factors, counting 11 million deaths and 255 million disability-adjusted life-years (DALYs), where 22% of all deaths and 15% of all DALYs were in adults aged 25 years or older. A diet based on the high consumption of sodium and the low consumption of fruit or whole grains was responsible for more than half of diet-related deaths and two-thirds of diet-related DALYs. Around the world, these results were consistent. Consequently, the influence of a suboptimal diet on NCD mortality and morbidity highlight the demand for improving diet habits across countries. GBD 2017 Diet Collaborators’ findings demonstrate that a substandard diet can be accountable for more deaths than any other risks worldwide, including smoking, which highlights the urgency for improving human diet around the world [7,12,13].

According to the World Health Organization, these premature deaths could have been prevented [9] through, for example, healthy eating habits [14], with the ingestion of fruits and vegetables which are the primary source of active phenolic compounds and some vitamins and may thus be a protection for population health [5,15,16]. Epidemiological studies have time and again demonstrated that a diet rich in fruits and vegetables, as well as whole grains, is intensely related to a reduced risk of developing NCDs [5,17,18,19,20].

The present review is aimed at explaining the beneficial effects of some food compounds such as phenolic compounds on physiologic processes that underlie the etiology of NCDs, such as inflammation and oxidation. Some mechanistic aspects are addressed for a better understanding of the process behind the appearance of inflammatory chronic diseases. An approach to specific examples of these diseases, rheumatoid arthritis (RA) and inflammatory bowel disease (IBD) was made in the context of inflammatory chronic diseases with the involvement of gut microbiota dysbiosis and impact on inflammatory responses. An exhibition of the scientific evidence from the impact of phenolic compounds on management of inflammatory chronic diseases (RA and colitis) will also be shown.

A comprehensive description of some features of phenolic compounds and some of their bioactivity descriptions in vitro and in vivo, and the beneficial health impacts on human health were presented. The phenolic compounds’ antioxidant and anti-inflammatory activities were more extensively described in this context. At this point, the discussion went through the gut microbiota modulation–immune system modulation axis by phenolic compounds. However, without forgetting that in addition to these compounds being able to modulate the gut microbiota, they are metabolized by microbiota, the bioavailability of these bioactive compounds was analyzed. This brought into the discussion the necessity of improving the limiting steps of this process for a more effective health impact with regard to dietary consumption and the actual larger demand for diet supplementation. In this context, some solutions that nanotechnology offers were presented, opening new doors for health prevention and treatment management of disease states.

## 2. The Impact of Oxidative Stress on Human Health

Several studies have demonstrated that inflammatory and oxidative processes take part in a considerable manner in the etiology of NCDs. It is commonly agreed that oxidative stress can trigger inflammation and that the inflammatory response (acute and chronic) generates excessive production of reactive oxygen species (ROS) and reactive nitrogen species (RNS) [21,22], thus entering into the maintenance of oxidative and inflammatory stresses. Acute inflammation as a defense mechanism of the body needs to be self-regulating because when uncontrolled it becomes chronic inflammation in which there is a persistent release of mediators that destroy tissues with pathological consequences for the body [23,24]. Unbalanced oxidative stress is responsible for the high production of free radicals that cannot be fully neutralized and removed by the body’s antioxidant system and can cause damage to biological macromolecules, contributing to the development of conditions such as cancer, autoimmune diseases, and type 2 diabetes (T2D) [25].

Molecular oxygen is fundamental in aerobic survival conditions; it is the final electron acceptor of the mitochondrial respiratory chain, which allows the formation of energy in the form of ATP, the re-oxidation of nicotinamide adenine dinucleotide (NADH) and flavin adenine dinucleotide (FADH_2_) [26]. ROS are formed in aerobic cells through respiratory chain electron transfer reactions and play important roles in biological processes [27] on physiological and pathological responses [28]. Some of its normal and important biological functions are related to energy production, phagocytosis, cell growth regulation and intercellular signaling and synthesis of biologically imperative compounds [29]. Under normal physiological conditions, the production of ROS is well controlled by performing its physiological functions without collateral damage [30]. Under stressful conditions, there is a decoupling of electron flow through the respiratory chain which can lead to unbalanced ROS formation [31].

Reactive oxygen species may originate from intracellular or extracellular sources through various sources such as mitochondria, peroxisomes (involved in fatty acid metabolism), lipoxygenases, NADH oxidase, and cytochrome P450 (electron transport chain microsome), among others [32,33]. Through the activity of cytosolic phospholipase A2 (cPLA2), metabolites are formed from arachidonic acid (AA) and ROS are released from membrane phospholipids [34]. Immunocompetent cells release nitric oxide (NO) and various oxygen radicals during a chronic immune response. There are also exogenous sources of ROS that include environmental drugs and toxins, air pollutants, cigarette smoke, UV radiation and heat shock [35]. In pathophysiological situations, excess ROS are produced as well, such as in a diet rich in polyunsaturated fatty acids, inflammation, ischemia/reperfusion, non-alcoholic fatty liver disease (NAFLD), rheumatoid arthritis, and cancer, as well as others [36,37,38,39].

The body has several antioxidant defense mechanisms against ROS: enzymes (catalase, superoxide dismutase, glutathione dismutase, glutathione peroxidase); endogenous molecules that can function as antioxidants (uric acid, glutathione, transferrin, ferritin, albumin, proteins that have –SH groups and bilirubin among others); non-enzymatic antioxidants are represented by l-ascorbic acid and *α*-tocopherol, glutathione (GSH), carotenoids, flavonoids, lipoic acid, arginine, citrulline, taurine, creatine, selenium, zinc and ubiquinone and other antioxidants [26,40].

Under regular conditions, stability exists between both activities and the intracellular levels of these antioxidants. This equilibrium is critical for the health and survival of organisms [31,41,42].

When free radicals are produced out of redox equilibrium or have not been neutralized by endogenous antioxidant defensive systems such as superoxide dismutase (SOD), catalase (CAT), peroxidase and glutathione peroxidase/glutathione system [30], the highly reactive capacity of ROS that characterize them react with biological molecules, such as DNA, tissue, enzyme proteins, cell membrane lipids and carbohydrates, inducing oxidation, which causes cell membrane damage, protein modifications (including enzymes), and DNA damage [26]. This oxidative stress generated from these imbalances, which ranges from ROS overproduction to enzymatic and non-enzymatic antioxidant deficits [31], can lead to the progress of conditions such as cancer, arteriosclerosis, gastric mucosal damage and degenerative aging processes [27].

However, antioxidant defense mechanisms in humans that are not completely efficient make it important to eat exogenous antioxidants to combat excess ROS [30,43]. Most dietary antioxidants are derived from eating vegetables, fruits, tea and wine, foods markedly rich in phenolic compounds [44,45]. The decrease in diseases, such as cancer, cardiovascular disease, amongst others, is attributed to the regular consumption of fruit and vegetables which contain natural antioxidants [46,47].

Phenolic compounds have been recognized as potent antioxidants for their ability to capture and trap free radicals and reduce other compounds, achieving greater antioxidant capacity than vitamin C and E [48,49]. Antioxidants, such as carotenoids and vitamins C and E, in a diet can reduce the risk of developing certain diseases [30]. Due to their chemical structure, phenolic groups of phenolic compounds may accept an electron or proton, forming relatively stable phenolic radicals preventing chain oxidation reactions in cell compartments [49]. Phenolic compounds act as antioxidants protecting human tissues against oxidative stress and conditions associated with this condition [50,51].

### 2.1. Inflammation in Human Health

Inflammation is a defense mechanism of the immune system where the body fights infections or aggressions from bacteria, viruses and other pathogens [52]. The clinical symptoms that define inflammation are known as flushing, heat, swelling, and pain [52].

Scientific knowledge about inflammation has evolved greatly since Celsus and Galen. Current knowledge about the inflammatory process and its implications for the onset of diseases has envisioned a new way of looking at this phenomenon. In addition, society in general today sees the phenomenon of inflammation as a major issue, which generates interest both in the scientific community and amongst the general public. This is because Time magazine in 2004 featured the issue of inflammation as its cover story, referring to it as the “Secret Killer” and described how this process is behind many common chronic diseases. The link between inflammation and diseases such as myocardial infarction, cancer, Alzheimer’s disease and others has come to everyone’s notice [53].

Just a few years ago, these diseases were not even related to the inflammatory process. Recently, new discoveries have attributed to the inflammatory phenomenon, an extremely important role in the pathophysiological processes of neoplastic, neurodegenerative, infectious, autoimmune and other diseases.

It is also known these days that pathologies, such as septic shock, hemorrhagic shock and multiple organ dysfunction, have long been considered to be of hemodynamic etiology, relating organ damage to simple lack of perfusion and tissue oxygenation, when nowadays this is just the tip of the iceberg and the triggered inflammatory process is the main cause of organ damage.

Inflammation rouses the immune response at the site of infection or injury, which is then stimulated by increases in vascular permeability and blood supply, which permits further penetration of plasma and leukocytes from the blood to the damaged tissues. This certain type of immune response is important since it aids the body repel pathogens and initiates the healing process of injured tissues, a reaction which is described as an acute inflammation [54]. During the inflammatory process, the cells release free radicals and reactive oxygen, and nitrogen species may enter in oxidative stress, causing cellular damage. These ROS and RNS are directly and indirectly involved in inflammation as they induce activation of transcription factors leading to the formation and release of chemical mediators such as tumor necrosis factor (TNF-α) and interleukins (ILs), which continue to stimulate the inflammatory process and the production of more free radicals. Thus, happens a cycle with amplification of the inflammatory response [55,56].

An inflammatory response is beneficial when self-limiting and involves inhibiting the expression of pro-inflammatory proteins, stimulating anti-inflammatory proteins, and reversing vascular changes that induce the initial process of immune cell recruitment [57].

Inappropriate or excessive activation, as well as failure to inactivate this defense mechanism, can have serious effects on human health [52]. These effects result in certain diseases or conditions in which the inflammatory response may be exaggerated or inadequate and maintained without any apparent benefit and even with adverse consequences, leading to chronic inflammation [58]. An example of this is ulcerative colitis (UC), which is a chronic and recurrent inflammatory disease in which there is inflammation of the colon mucosa and whose incidence in Europe is 5–25 new patients per 100,000 individuals per year [59]; furthermore, it often evolves into colon cancer. Similarly, rheumatoid arthritis is another chronic inflammatory disease whose prevalence is increasing and reaching 1% of the world’s population [60].

Studies have demonstrated that chronic inflammation is a progenitor of tumor progression; many cancers have been found to rise from sites of infection, chronic irritation and inflammation. Inflammatory cells and the signaling molecule network provided by the inflammatory microenvironment are required for malignant progression of mutated/transformed cells [54]. According to several works, cancer can be seen as the consequence of a failure in response, essentially oriented to wound healing. In this perspective, inflammatory mediators offer a wide range of potential targets for therapeutic or preventive interventions [61,62].

Chronic diseases, such as cancer, lung and cardiovascular diseases, neurological diseases, diabetes and autoimmune diseases, have the same picture of increased inflammatory response, which is often observed at a very early stage of the condition, even before being diagnosed [52,63,64].

For over 100 years, there has been evidence of inflammation’s involvement in diabetes, when high doses of salicylates have been shown to lower glucose levels in diabetic patients [65,66].

Elevated levels of inflammation markers and mediators and acute phase reagents such as fibrinogen, C-reactive protein (CRP), interleukin-6 (IL-6), plasminogen activator inhibitor-1 (PAI-1), sialic acid, and leukocyte count correlate up with the incidence of T2D [67,68,69,70,71,72]. Levels of inflammation and coagulation markers can be reduced with intensive lifestyle intervention, as was the case in the diabetes prevention program published by some authors [73], but there are experiments showing that pro-inflammatory cytokines, such as tumor necrosis factor (TNF-α), may derive from adipose tissue and provoke insulin resistance in experimental models. This evidence gave the necessary impetus to start thinking in terms of pathogenesis [74,75,76].

Different areas of research have come together enough that inflammation can now be linked to the development of insulin resistance and the pathogenesis of T2D [77,78]. In fact, the expansion of the concept of insulin resistance and T2D having immune components and a better understanding of how inflammation shapes metabolism offers new prospects for using anti-inflammatory strategies to right the metabolic costs of adipocyte excess [79]. It is unknown if ROS can contribute towards maintaining insulin sensitivity in vivo through the inhibition of protein tyrosine phosphatases (PTPs). Loh and colleagues present evidence for the enhancement of insulin signaling by ROS in vivo where they showed that mice missing a crucial enzyme [glutathione peroxidase 1 (Gpx1)], necessary for the neutralization of ROS physiologically, were guarded from insulin resistance induced by a high-fat diet. The increase in Gpx1^−/−^ mice insulin sensitivity was associated with phosphatidylinositol-3-kinase (PI3K)/Akt signaling induced by insulin, and glucose uptake in muscle and could, by the antioxidant *N*-acetylcysteine, be inverted. An increase in insulin signaling was associated with higher oxidation of phosphatase and tensin homologue (PTEN, a PTP family member), which terminates PI3K created signals. Glucose turnover and insulin sensitivity were determined in high-fat-fed weight-matched Gpx1^−/−^ versus Gpx1^+/+^ male mice by execution hyperinsulinemic euglycemic clamps. As calculated by the infusion rate of glucose during hyperinsulinemic-euglycemic clamps, insulin sensitivity was augmented by about 3-fold in Gpx1-deficient mice. Additionally, insulin-stimulated glucose disappearance, which primarily reveals skeletal muscle glucose disposal, was augmented in Gpx1^−/−^ mice, while hepatic glucose production was not changed. The researchers discovered no clear difference between Gpx1^−/−^ and Gpx1^+/+^ mice in the expression of hepatic gluconeogenic gene as determined quantitatively, while the uptake of 2-deoxy-[1-^14^C] glucose was increased by close to 3 to 4-fold in Gpx1^−/−^ diaphragm skeletal and white gastrocnemius muscles. These results indicate altogether that insulin sensitivity is raised as a consequence of a deficiency of Gpx1 and that this may be endorsed to the increase in muscular insulin-induced glucose uptake. It was then investigated if the raised ROS was accountable for the enhancement of insulin sensitivity and PI3K/Akt signaling. For 7 days, male versus female Gpx1^−/−^ versus Gpx1^+/+^ mice fed a high-fat diet were served the antioxidant *N*-acetylcysteine (NAC (a H_2_O_2_ scavenger and motivator of glutathione production)). NAC decreased muscle H_2_O_2_ and PI3K/Akt signaling in −/− mice to levels seen in Gpx1^+/+^ mice. Additionally, administration of NAC augmented fasted blood glucose in Gpx1^−/−^ mice and rendered Gpx1^−/−^ mice more insulin resistant so that they looked similar to Gpx1^+/+^ mice while lacking any obvious impact on body weight. NAC did not affect Gpx1^+/+^ mice insulin sensitivity. These outcomes deliver causal evidence for ROS involvement in the increase in insulin sensitivity/signaling in Gpx1^−/−^ mice fed a high-fat diet in vivo. As compared to their wild-type, Gpx1^−/−^ mice remained more insulin sensitive. These outcomes are thus in line with the rise in insulin sensitivity in Gpx1^−/−^ mice independently of body weight, and for the rise in muscle insulin responsiveness as a potential factor for the lean phenotype [80].

Doses of natural product-based supplementation are an extremely important health issue as demonstrated by research studying the effects on insulin sensitivity. For this propose, the study combined vitamin E (400 IU/day) and vitamin C (1000 mg/day) supplementation, and nineteen untrained participants and twenty pre-trained healthy young men were tested before and after 4 weeks of physical exercise practice. The achieved results revealed that physical exercise had the effect of elevating the insulin sensitivity parameters (plasma adiponectin) only where antioxidants in both previously untrained and pretrained individuals were absent. These results were accompanied by an augmentation of ROS defense capacity and in the expression of regulators of ROS-sensitive transcriptional of insulin sensitivity, peroxisome-proliferator-activated receptor gamma (PPARγ), and PPARγ coactivators, PGC1β and PGC1α only, in the nonexistence of antioxidants. Molecular mediators of endogenous ROS defense (SOD 1 and 2; GSH) were also exercise-induced, an effect that was also blocked by supplementation of antioxidants. True to the concept of mitohormesis, oxidative stress induced by exercise improves resistance to insulin and creates an adaptive response which promotes endogenous antioxidant defense capacity [81]. The effects of exercise on health promotion in humans might be precluded by antioxidant supplementation. Clearly more studies surrounding this issue must be developed, since it is clear that redox homeostasis, comprised of multiple redox circuits, involves sensing and adapting to stress. This mechanism, called mitohormesis, is responsible for eliciting helpful adaptations that can reinstate redox homeostasis and help secure skeletal muscle from oxidative damage and mitochondrial dysfunction. With age, redox homeostasis is compromised, which leads to mitochondrial oxidative damage and skeletal muscle dysfunction. Complementary or alternative interventions are essential to preserve or reestablish redox homeostasis as a means to preserve skeletal muscle function and health span [82].

The perseverance of inflammation in a chronic form creates a profoundly altered environment that favors the incidence of transformed cells and their development into malignant cancers [62,83]. Chronic inflammation is characteristic of most solid tumors and is an antecedent to a wide variety of cancers including, but not limited to, colon, esophagus, stomach and bladder cancer [61]. Chronic inflammation is defined by tireless attraction of immune cells that secrete precise mediators at the site of injury [61]. These mediators include DNA-damaging free radicals, chemokines, and cytokines, which reduce apoptosis, bolster neo-angiogenesis, boost cell proliferation, stimulate stromal cell development, and enable cell motility and invasion [61,62]. Cytokines are created in a wide range of intersecting biological effects, functioning within a multifaceted signaling network [61,62]. Of particular importance in cancer are IL-1 and 6, TNF-α, chemokines and their receptors, as well as death domain family receptors [61]. Resident cells from inflammatory sites adapt by producing factors that increase their survival [61]. One such factor is cyclooxygenase-2 (COX-2), which is controlled by the interaction between “tumor suppressor protein 53” (p53) and nuclear factor kappa B (NF-κB)—two factors with broad roles in inflammatory responses [61].

Pro-inflammatory states can still be achieved at the expense of lifestyle in modern societies [84]. Eating habits that include consuming high-calorie diets rich in sugars, saturated fatty acids and trans fats give rise to a persistent pro-inflammatory state [84,85,86], in which circulating levels of pro-inflammatory cytokines, neutrophil recruitment and oxidative stress increase [84,85]. Interaction between said pathways perpetuates a feedback process in which an inflammatory state increases the risk factors for various diseases.

Another relevant factor in the impact of inflammatory conditions in the health of populations is longevity in modern societies. Given the major demographic trends in almost all of Europe, it should be noted that in countries such as Portugal, for instance, which is the sixth oldest country in the world, since 2010 it is one of the few European countries with both negative (natural and migratory) balances. By 2051, the Portuguese population is estimated to be 8.4 million (lower than in 1950); it will be difficult to prevent the decline of the population where the number of the elderly is almost directly proportional to the decrease in youth. By 2051 there will be three times more the amount of older people in relation to younger people, the adult population will have an older average age and the very old group (85 or older) will be three times older. All of this will cause negative effects potentiated by fiscal pressure on the Social Security System [87], Healthcare System and others.

**Figure 1:**
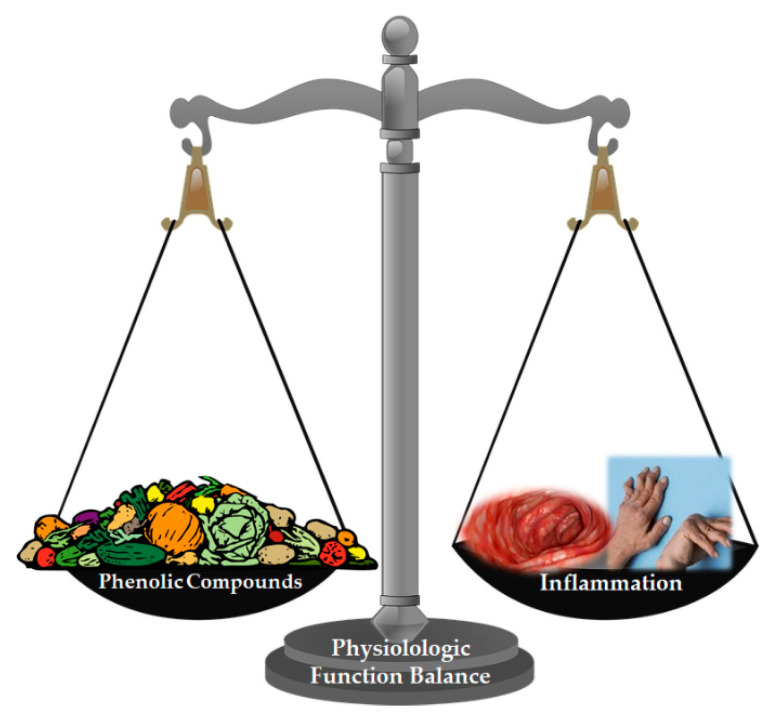
Most dietary antioxidants are derived from eating vegetables, fruits, tea and wine, foods markedly rich in phenolic compounds. The antioxidant defense mechanisms in humans that are not completely efficient make it important to eat exogenous antioxidants to combat excess ROS which can lead to the progress of conditions such as cancer, rheumatoid arthritis, inflammatory bowel disease, etc.

Given this scenario, studies involving older humans made the study of inflammation in this population pertinent. A recent study of 1554 very old individuals (100–104 years old), semi-super-centenary years old (≥105 years old), 85–99 years old and their descendants, showed, for example, that the length of telomeres was not a predictor of aging in centenarians and semi-supercentenarians (they were able to maintain long telomeres), but that inflammation is an important malleable device of aging to extreme advanced age in humans [88]. The results achieved by Arai et al., in 2015, suggest the subduing of chronic inflammation as an important aspect behind successful longevity, an aspect pertinent over a very wide age range to extreme advanced age. Despite the limitations, the study showed that over a very large age range of 45 to 115 years, including an unprecedented number of extreme age individuals, inflammation is an important aging engine that may be useful for future pharmacological intervention. Consequently, the design of a new and safe anti-inflammatory or immunomodulating drug has potential for contributing to a healthy life [88]. In the meantime, supplements and diets with anti-inflammatory potential, studied for this purpose in this group of the population, may be the key to greater longevity and above all healthier lives. It is a strategy where prevention rather than treatment can be used. One of the widely recommended preventive measures is to increase the consumption of fruits and vegetables, naturally rich in bioactive compounds that may contribute to reducing the risk of suffering from diseases associated with oxidative stress, due to the wide variety of antioxidant compounds that these foods contain [84,85,89]. This antioxidant effect is mainly due to the phenolic compounds present in these foods [84,85] but also the presence of some antioxidant vitamins [90,91]. There is great potential for dietary phenolic compounds to become the next generation of health-affecting dietary factors in inflammation control, in addition to synthetic drugs that already exist for this purpose. These compounds can also provide an excellent model for the development of more effective and safe future chemopreventive compounds (Figure 1) [52,92].

#### 2.1.1. Rheumatoid Arthritis (RA)

Rheumatoid arthritis is a multifactorial disease encompassing genetic and environmental factors [60]. This is one of the most representative examples of chronic autoimmune inflammatory disease, partly triggered by T lymphocyte activation and release of cytokines, interleukins and TNF-α [93]. Deregulation in both innate and adaptive immune mechanisms lead to the production of autoantibodies and dyslipidemia, which may precede the start of clinical disease by up to a decade [94].

Rheumatoid joints consist of an uncommon pathophysiological environment made up of hypoxia and variable biomechanical stress that can command immune system signaling pathways [95]. Unbalanced generation of ROS in damaged joints accelerates inflammatory responses in RA patients [96]. The cytokines produced take part in the immunoregulatory and tissue destruction events that are behind the clinical exhibition and progression of rheumatoid arthritis [95].

Monocyte and lymphocyte migration in the synovial membrane of rheumatoid arthritis is mediated by abnormal expression of various adhesion molecules (ELAM-1, VCAM-1, ICAM-1, ICAM-2) [97] which can be explained by the abnormal induction of redox-sensitive signaling pathways [31]. In joints with RA, various inflammatory cells including adaptive immune cells (B and T cells), innate immune cells (e.g., dendritic cells, natural killer cells, mast cells, and macrophages), and synovial fibroblasts (fibroblast-like synoviocytes (FLS)) are enabled. These cells interact with each other through a succession of cytokines and/or cell-to-cell contacts, which lead to abnormal proliferation of FLS, continued inflammation, and destruction of bone and cartilage [98,99,100]. The intense neovascularization process with associated lymphangiogenesis facilitates intense recruitment of all immune cells [94]. It is also acknowledged that host tissue cells (osteoclasts, chondrocytes, and activated synovial fibroblasts) are involved, mediating bone and cartilage destruction as well as stimulating the perpetuation of inflammation [94].

Severe synovial membrane inflammation (synovitis), where there is a 3–100-fold elevation of pro-inflammatory cytokines, such as TNF-α, interleukin-6 (IL-6), interleukin-1β (IL-1β), and CRP [101], leads to chronic synovitis [94]. Chronic inflammation of the synovial membrane and B and T lymphocyte dependent immune reactions, together with synovial membrane hyperplasia, lead to the formation of *pannus*, a tissue composed of immune cells, blood vessels (angiogenesis) and fibrous cells [95]. Macrophages play an extremely important role in the injury caused by rheumatoid arthritis due to their abundance in the synovial membrane and the cartilage-*pannus* junction. Overexpression of major histocompatibility complex (MHC) class II, pro-inflammatory or regulatory cytokines and growth factors (e.g., IL-1, IL-6, IL-10, IL-13, IL-15, IL-18, TNF-α, and GM-CSF), indicate to us that these cells are clearly activated. Although macrophages are not the cause of the disease, they have remodeling, destruction and pro-inflammatory capabilities that contribute significantly to joint inflammation and destruction in both acute and chronic phases [102].

*Pannus* proliferation occurs early in the disease and can be seen before cartilage and bone destruction [103]. Cartilage destruction is mediated by matrix metalloproteases (MMPs) synthesized in activated macrophages and pro-inflammatory cytokine-stimulated fibroblasts (i.e., IL-1 and TNF-α). The most important MMPs in RA are collagenase (MMP-1) and stromelysin 1 (MMP-3) [104]. These metalloproteases are capable of degrading structural proteins of the cartilage extracellular matrix. This erosion may be caused by increased expression of receptor activator of nuclear factor kappa-Β ligand (RANKL) and macrophage colony stimulating factor (M–CSF) which are indispensable for osteoclast differentiation [105].

The contents of neutrophil granules such as myeloperoxidase, elastase, metalloprotease and collagenase cause tissue damage and amplify neutrophil response. On the other hand, activated neutrophils are also capable of releasing cytokines, such as TNF-α, IL-1, IL-6 and transforming growth factor β (TGFβ). These cytokines will affect the neutrophils themselves and other cells [106]. Finally, chondrocytes move from an anabolic state in which they synthesize matrix to a catabolic state with the release of metalloproteases and cytokines, namely, IL-1β, Il-17, IL-18 and TNF-α, thus contributing to degradation of cartilage [95].

This results in bone and cartilage degeneration and pain, which can lead to severe, temporary or permanent functional disability, or even death, in the most advanced conditions of the disease [107]. The most affected tissues are the synovial membrane, cartilage and bone [95]; however, it may have systemic implications, increasing for example the risk of coronary heart disease [108].

Circulating cytokine levels reflect disease activity and the degree of inflammation present and may also play a significant role in systemic effects of diseases such as vascular disease [108] and rheumatoid cachexia [109]. The development of rheumatoid cachexia is associated with overproduction of pro-inflammatory cytokines, in particular TNF-α and IL-1β, accelerated protein catabolism, and poor physical activity [110,111], which, in turn, predisposes these patients to be physically inactive and more likely to be obese or overweight [112]. This condition increases the risk factor for metabolic syndrome [113], also contributing to systemic inflammatory responses [114]. Cardiovascular disease accounts for about 40% of deaths in rheumatoid arthritis patients; followed by cancer (17%), infections (14%), musculoskeletal disease (9%), respiratory disease (9%), and kidney disease (9%) [115].

Therefore, RA results in considerable direct costs such as health costs, and indirect costs such as lost productivity due to morbidity and decreased life expectancy [116].

Therapeutically, acetylsalicylic acid and other non-steroidal anti-inflammatory drugs (NSAIDs) have long been administered to fight inflammation [52]. Cyclooxygenase-2 (COX-2) inhibitors have been suggested for the treatment of RA and osteoarthritis [52], although some of these were ultimately withdrawn from the market due to unforeseen side effects [52].

Modern approaches to treatment using agents such as methotrexate and biological agents have revolutionized treatment, but the disease is still progressive, with long-term joint damage [117]. Methotrexate is a folic acid antagonist that is widely prescribed at low doses to treat RA, psoriasis [118,119], and chronic inflammatory bowel disease [120]. This treatment is effective, and the main limiting factor for its use is toxicity; gastrointestinal side effects in particular are the main reason for discontinuation [118,119]. A recent case reported that a 40-year-old man receiving chronic methotrexate therapy had severe apoptotic enteropathy with hypovolemic shock and watery diarrhea which came as a result after switching from oral to intramuscular route without any dosage changes. Colon biopsies proposed drug-induced colitis, demonstrating a mild non-specific inflammatory infiltrate of lymphocytes and plasma cells, damaged and dilated crypts, and a marked rise in basal crypts apoptosis [121].

However, given that conventional therapeutic and surgical methods have not been able to fully govern the occurrence and outcome of many inflammatory diseases, there is a critical need to find new and safe compounds as an alternative to approaches in the management of these diseases [52].

A systematic review has suggested that there is evidence of a protective effect from increased fruit and vegetable intake in the development of RA [122,123]. Dietary antioxidants effectively suppress inflammatory cytokine release by in vitro studies [124,125,126,127,128]. The Mediterranean diet model has previously been associated with health benefits. Specifically, the fat and non-fat components of the Mediterranean diet pattern (MDP) have been demonstrated to exert significant anti-inflammatory activities by impacting the action of immune cells, the manifestation of some pro-inflammatory genes, and the arachidonic acid cascade [129,130].

Infiltrating leukocytes such as monocytes, synoviocytes, and neutrophils are critical sources of eicosanoids in rheumatoid arthritis [131]. The effectiveness of NSAIDs, which act to constrain COX activity in RA, indicates how critical this pathway is in the pathophysiology of the disease. A number of pharmaceutical agents used as anti-inflammatory agents act on the COX pathway [23]. It has been observed that phenolic compounds have an obstructive effect on protein expression in relation to COX-2 and metalloproteinase and prostanoid production [132], which are known to play an important part in angiogenesis, a chief pathogenic mechanism linked to atherosclerotic vascular diseases, chronic inflammatory joint diseases, but also cancer [128,129,133]. Genistein is abundant in soybeans and has gained attention due to its possible role in preventing and treating several disorders [134,135]. Genistein (4′, 5, 7-trihydroxyisoflavone) is a flavonoid which occurs naturally, distinctive of *Leguminoseae* plants. This flavonoid is a phyto-estrogen exerting estrogenic activity as substance that is both an agonist and an antagonist. Much research indicates that genistein has numerous pharmacological and physiological characteristics which mark this molecule as a possible agent for the treatment and prevention of several chronic diseases [136]. Genistein appears to have significant anti-inflammatory and immunoregulation effects [134,135], and it seems that it can inhibit angiogenesis and relieve inflammation in the collagen-induced arthritis (CIA) animal model while lowering the expression of IL-6 and VEGF [137,138]. Additionally, genistein has also shown an ability to constrain VEGF expression and angiogenesis in tumors [139,140] The role that the overactivation of STAT3 and overexpression of VEGF play cannot be ignored during angiogenesis [141,142,143,144] and researchers have seen that IL-6 augments the expression of VEGF and angiogenesis by activating STAT3 in tumors [145,146,147]. Upregulation of several angiogenic and pro-inflammatory mediators orchestrates the characteristic pathological synovial alterations in rheumatoid arthritis [148]. Research demonstrates that angiogenesis can be hampered under an environment of inflammation as well as impede the inflammation-induced expression of VEGF in MH7A cell partly through the pathway IL-6/JAK2/STAT3/VEGF, providing new prospects for treating rheumatoid arthritis [148]. Results demonstrated that genistein could impede angiogenesis and that angiogenesis was inhibited partially through the signaling pathway JAK2/STAT3/VEGF, providing a fresh insight into the antiangiogenic activity of genistein in rheumatoid arthritis, for which genistein may be a suitable candidate to develop a new drug for treating this disease [148].

While studying the isoflavone genistein, Cepeda and colleagues showed evidence that genistein significantly increased extracellular collagen deposition and osteocalcin expression. In primary cell cultures of calvarial, osteoblasts isolated from female Wistar rats that had been exposed to genistein in vitro showed that genistein stimulated osteoblastogenesis through the participation of the estrogen receptor and NOS pathways, as well as the influence of ERK or PI3K signal transduction pathways; additionally, genistein also stimulated osteoclast differentiation from its progenitor mononuclear. Even though these results were obtained from in vitro assays, they provide insights to enlarge the knowledge about genistein contribution on bone homeostasis maintenance [149]. In this aspect, Xu and colleagues contribute to this issue by exploring genistein’s anti-angiogenic effects on synovium in a type II collagen-induced arthritis rat model. Their results showed that genistein lessened levels of MMP-1, 2 and 9 and vascular endothelial growth factor (VEGF) and in CIA rats’ sera. Microvessel density in synovium in treated groups was lowered when compared to the control group and the effects of genistein administered with methotrexate are preferable to single agents in treating RA [150].

The therapeutic value of resveratrol in RA was tested on humans to explore the influence of resveratrol oral capsules (1 g of resveratrol), added to conventional RA therapy, on the clinical and biochemical markers in 100 rheumatoid arthritis patients through a randomized controlled clinical trial. Each member of the test group received one daily oral soft gel capsule enclosing 1 g of resveratrol during 3 months in addition to the disease-modifying anti-rheumatic drugs (DMARDs) each patient was receiving, while members of the control group received just their conventional treatment. The effect of a daily single oral dose of 1000 mg of resveratrol co-administered with conventional anti-rheumatic drugs to RA patients over a period of 3 months, compared with the control group, the resveratrol-treated group has shown significant drop in the key clinical and biochemical markers involved in the disease’s activity mechanism. These indices remained almost unchanged in the control group throughout the duration of the protocol. The achieved results have shown a significant effect of resveratrol in lowering the serum levels of TNF-α and IL-6 [151], which was reported to be significantly higher in the RA population than in healthy people [152]. In this study, the patients have safely tolerated the resveratrol dose administered without reported adverse events [151].

Resveratrol’s mechanism functions by inhibiting MAPK signaling pathways, possibly by decreasing the accumulation of ROS, in order to suppress cell proliferation and the inflammatory response and to provoke cell apoptosis in the synovial tissue, along with mitigation of HIF-1α-mediated angiogenesis. As such, resveratrol appears to hold great potential for clinical translation as a novel therapy for RA, as showed by an in vivo study with bovine type-II collagen (BIIC)-induced Sprague–Dawley rat arthritis model and an in vitro arthritis model based on interleukin (IL)-1β-stimulated rat synovial cells (RSC-364) [153]. Resveratrol alleviated arthritis through the activation of Nrf2-ARE (antioxidant response elements) signaling pathway via SIRT1/NF-κB/miR-29a-3p/Keap1 and SIRT1/NF-κB/miR-23a-3p/cul3 signaling pathway [154]. Stimulation by resveratrol of Sirt1 suppressed COX-2/PGE2 production by hindering the interaction of AP-1 and NF-κB in rheumatoid arthritis synovial fibroblasts. Resveratrol stifled the acetylation and phosphorylation of p65, Fos, and c-Jun, and decreased binding to the COX-2 promoter, which diminished COX-2 expression [155]. The inner workings of the pro-apoptotic and antioxidant impacts of resveratrol in H_2_O_2_-treated arthritis fibroblast-like synoviocytes (RA-FLSs) by the signaling pathway Nrf2–Keap1 were studied by Zang and colleagues. The research team discovered that resveratrol constrains the production of ROS by stimulating the Nrf2 pathway, impeding NF-κB activation and migration and proliferation of RA-FLSs, to promote apoptosis [156]. Resveratrol was also found to ameliorate the damage and swelling and lowered MMP1 and MMP13 expression levels in CIA rats [157]. Resveratrol was found to inhibit STAT3, Src kinase, and Wnt signaling pathways active in the CIA model, thereby improving inflammatory arthritis [158].

The combination therapy utilizing methotrexate with the food component in the transdermal delivery system was investigated and evaluated for its anti-inflammatory and anti-arthritic potential. Transdermal gel containing methotrexate-resveratrol loaded nanoemulsions was used in order to survive bioavailability and detrimental impacts of RA monotherapy, and, as consequence, potential anti-arthritic and anti-inflammatory activities of the combination in nanocarrier were tested in rats, demonstrating 78.76 ± 4.16% inhibition in inflammation as well as improved anti-arthritic effects. Integrating dual delivery with nanotechnology can produce potentially successful options for treating rheumatic diseases [159]. The combination therapy is finding wide application for enhancing the bioavailability and therapeutic effects of the individual drugs [160]. Thus, future preclinical studies are warranted for moving this novel treatment modality from bench to bedside [159].

Even though some aspects of the mechanisms of action by which the MDP exerts its beneficial effects have yet to be elucidated, arthritis patients may potentially benefit from it, given its increased cardiovascular risk and the treatment that may have side effects [129,130].

#### 2.1.2. Inflammatory Bowel Disease (IBD)

The intestine is also a target organ of chronic inflammation. The worldwide incidence and prevalence of IBD has increased dramatically over time, highlighting its emergence as a global disease [161,162]. Chronic onset inflammation typically appears via two diseases, Crohn’s disease (CD) or ulcerative colitis (UC) [163]. They manifest themselves through ulcers, some very severe in the intestines. These diseases affect about 10% of the world’s population [164] with gastrointestinal symptoms such as bloody diarrhea, abdominal pain, anemia, and weight loss. The most common form of inflammatory bowel disease worldwide is ulcerative colitis [165]. Northern Europe and North America have the highest prevalence and incidence of ulcerative colitis: prevalence rates of 156–291 cases per 100,000 people and incidence ranges of 9–20 cases per 100,000 person years. Rates tend to be lower in eastern countries and in the southern hemisphere. Incidence has risen in countries that have embraced an industrialized lifestyle, suggesting that environmental factors may be critical in triggering the disease [166]. The onset of IBD is associated with smoking, high fat and sugar diets, medication use, stress and high socioeconomic status [167].

A westernized diet, a diet typically high in sugar and animal fat whilst low in fiber, has been suggested as a risk factor for inflammatory bowel disease development. These theories result from the incidence of IBD in places such as in Asia and in Eastern Europe, where westernized diets have seen a rise in adoption [168]. Various researcher groups have reported on associations between various foods such as fast food, margarine, refined sugars, cornflakes and some dairy products. There is no certain evidence, however, to indicate a direct contribution of any specific dietary factor to the development of inflammatory bowel disease [169]. NSAIDs and infections can transiently trigger nonspecific inflammation, break down the mucosal barrier and stimulate innate immune responses. These events may then lead to an increased uptake of commensal and adjuvant bacterial antigens that motivate prolonged T cell-mediated intestinal inflammation in the genetically susceptible host [170].

The exact etiology of IBD is unknown. It is generally agreed, however, that a complex interaction between an environmental depletion, an infectious environment, an aberrant immune response, and genetic predisposition all contribute to the onset and progression of the disease [171,172,173]. Research indicates that inflammatory bowel disease can arise from an abnormal immune response to a genetically susceptible host’s intestinal bacteria [174]. Molecular studies have characterized a series of genetic determinants for the susceptibility to IBD development and variants that may govern disease phenotypes [175]. Genetic irregularities of this sort might be a possible reason for the development of defects in epithelial barrier function, bacterial removal and immunoregulation [174], which will interrupt proper intestinal immune system regulation [176]. Transcytosis is the mechanism by which macromolecules are transported through enterocytes. A recent study points to the role played by dysregulated transcytosis as an inflammatory response initiator by increasing exposure to intestinal lumen antigens [177]. In Crohn’s disease (CD), it is the combined effects of Interferon-gamma (IFNγ) and TNF-α that lead to epithelial injury, leading to a reduction in the number of connections at the tight junctions, breaks in them and alteration of protein content and composition of these junctions. Altogether, these alterations lead to the development of diarrhea by a leakage flow mechanism and uptake of lumen antigens [178].

Mortality in IBD is low [179], diagnosis is mostly made while patients are still young [180], it is thus predicted that the global prevalence of IBD will continue to increase substantially over the coming decades. However, the ultimate reasons for rising IBD rates are largely unknown [161]. Even though the etiology of IBD has been extensively studied in recent decades [181], the pathogenesis of the disease is not yet fully understood [182,183].

In CD, selenium and glutathione peroxidase activity are decreased and this decrease is inversely related to TNF-α levels and erythrocyte sedimentation rate [184]. With no difference between CD and UC, both vitamin E and vitamin A levels are decreased [185]. It was previously thought that it was only Th1 lymphocytes that initiated and perpetuated CD, but a new lymphocyte subtype, Th17, has also been discovered that is also involved in other inflammatory diseases such as rheumatoid arthritis [95,186].

Despite the similar impact that oxidative stress has on both diseases, there are some distinct parameters. For example, in both CD and UC, malondialdehyde is increased, however, in CD it is associated with metallothionein and manganese-dependent superoxide dismutase and in UC it is associated with catalase and glutathione peroxidase. From there data, the researchers came to the conclusion that, in CD, oxidative damage will be more related to the presence of hydroxyl radicals and superoxide anions, while in UC, hydrogen peroxide and/or hypochlorous acid would be the main agents [187].

As usually happens with inflammatory reactions, there is a release of oxidative species. In addition, in UC, the injury can be caused by oxidative stress. In UC, high levels of DNA oxidation products (i.e., 8-hydroxy-2’-deoxyguanosine) [188], ROS [189] and iron in inflamed tissue [190] have been found. Antioxidant defense mechanisms are diminished, which may be due to an excessive inflammatory response. This is demonstrated by the decrease in superoxide dismutase, the enzyme in charge of catalyzing the conversion of superoxide to oxygen and hydrogen peroxide [189].

Given the high incidence of IBD in western countries, its clinical treatments have been shown to cause major side effects. They are costly and their objectives are ambiguous, and the healing effects are not satisfactory [191]. Pharmacological treatment for IBD has progressed from a conventional treatment with low-selectivity aminosalicylates, immunosuppressants and corticosteroids, to a new generation of biopharmaceuticals, primarily monoclonal antibodies (mAb) targeting TNF-α, mainly in moderate to severe disease-resistant manifestations to conventional therapies. There is an increased risk for malignancies, however, and a loss of therapeutic response over time due to the development of therapeutic mAb antibodies [192].

Methotrexate toxicity includes rash, nausea, diarrhea, mucositis, hypersensitive pneumonitis, bone marrow suppression, infection, elevated transaminases, and liver fibrosis or cirrhosis [193]. Cyclosporine and tacrolimus toxicity includes hypertension, headaches, paresthesias, convulsions (cyclosporine only), gingival hyperplasia (cyclosporine only), hypertrichosis (cyclosporine only), diabetes mellitus (tacrolimus only), anaphylaxis (cyclosporine only), infection (sepsis and opportunistic infections), and renal failure [193].

Toxicities of anti-TNFα antibody (i.e., adalimumab, certolizumab pegol, and infliximab) include: infusion and delayed type hypersensitivity reactions (infliximab only), self-formation antibodies (mainly infliximab), injection site reactions (adalimumab and certolizumab pegol only), demyelinating (optic neuritis, multiple sclerosis), drug-induced lupus, reactivation of latent tuberculosis, worsening of congestive heart failure, severe infections (both sepsis and opportunistic infections), non-Hodgkin’s lymphoma, and possibly malignant diseases such as solid tumors [193,194,195].

A therapeutic demand therefore remains for a pharmacological method that weakens the progression of inflammatory processes in the colon, with a lower incidence of adverse effects. Natural anti-inflammatory medicines have great efficacy and low toxicity making them desirable for treating IBD patients [191,196].

Some studies are emerging in this field, which try to clarify the reflection of the use of some foods present in the Mediterranean diet and their effect on the development of this disease in the populations where they are consumed. An example is extra virgin olive oil, which is a dietary fat characteristic of Mediterranean cuisine, which has been tested in a chronic colitis model. The results showed that it exerted a remarkable beneficial effect by cytokine modulation and the reduction of COX-2 and inducible nitric oxide synthase (iNOS) by decreasing p38 MAPK expression. Together with the high proportion of oleic acid, many of these benefits are due to the high content of phenolic compounds [197]. Spearmint (*Mentha spicata* L.) is a Mediterranean plant used as a cuisine aromatic agent exhibiting acute and chronic in vivo anti-inflammatory activity with reduction in colon lesion and inflammation, reduction in histological markers and reduction of iNOS expression [198]. Another Mediterranean aromatic plant, which is used extensively as a spice in gastronomy and as a food preservative by the food industry, is Pennyroyal (*Mentha pulegium*). Although not much is known regarding the pharmacological effects of pennyroyals’ phenolic compounds, the decrease in several markers of colon inflammation was observed after orally administering a phenolic extract to colitis-induced mice, including histological and functional indicators. This extract also led to a diminished expression of iNOS and COX-2 in the colon of colitis-induced mice, both of which are vital mediators of intestinal inflammation [199].

The administration of mango pulp (*Mangifera indica* L.) as an adjuvant treatment combined with the conventional use of medications in patients (for 8 weeks, ten participants with mild to moderate IBD received a dose of 200–400 g of mango pulp daily) showed to have beneficial results. Mango intake lowered levels in the plasma of pro-inflammatory cytokines, which includes granulocyte macrophage colony-stimulating factor (GM-CSF), growth-regulated oncogene (GRO), and interleukin-8 (IL-8), and improved considerably the primary outcome Simple Clinical Colitis Activity Index score [200].

Maqui berry (*Aristotelia chilensis*) extract was tested in dextran sulfate sodium (DSS)-induced ulcerative colitis mice where results from experiments demonstrated that the ethyl acetate fraction of maqui berry water extract was rich in phenols and exhibited good anti-inflammatory and antioxidant activities. The inflammatory bowel disease index, i-NOS, NO, MDA, and COX-2 in colon tissues and TNF-α, MPO, and IL-1β in blood serums, were decreased in the treatment group as compared to the model group. The treatment group also saw a significant mitigation of intestinal histopathological damage. The lack of equilibrium caused by DSS injury on gut microbiota was alleviated by the treatment with Maqui berry water extract [201].

Some research has demonstrated possible anti-inflammatory effects obtained from several by-products of the industrial extraction of orange juice in mice with DSS-induced colitis. Fresh and dry orange residue, animal feed, and orange liqueur, not to mention commercial citrus pectin, were administered for 15 days to C57BL/6J mice before starting DSS treatment. An analysis performed of several macroscopic parameters such as the colonic weight/length ratio, and the Disease Activity Index, revealed that an anti-inflammatory effect could be found following the consumption of fresh orange residue, animal feed, or citrus pectin. Quantitative polymerase chain reaction (q-PCR) of RNA from colonic tissue demonstrated measurable alterations in the expression of iNOS, IL-1β, TNF-α, and intercellular adhesion molecules ICAM I, not to mention intestinal barrier proteins just as ZO-1, occludin, and MUC-3. Phenolic compounds, pectin and/or Maillard reaction products, formed at initial steps, have been identified as relevant components that exert the attributed beneficial effects [202].

Treating 2,4,6-trinitrobenzene sulfonic acid (TNBS) colitic rats with an ethanolic extract of *Terminalia catappa* stem bark produced a decrease in weight/length ratio and in the colonic damage score. Colonic neutrophil infiltration decreased as well, designated by a decrease in the activity of myeloperoxidase and interrupted the reduction of colonic glutathione levels in colitic rats. Treating with ethanolic extract of *Terminalia catappa* stem bark down-regulated gene expression of iNOS and pro-inflammatory mediators (IL-6, IL-23, TNF-α and CINC-1) in colitic rats. Furthermore, the gene expression of mucosal barrier proteins such as villin, MUC-2, and MUC-3 were stimulated in colitic rats treated with ethanolic extract of *Terminalia catappa stem bark*. The most significant beneficial impact was produced by the ETCB dose of 100 mg/kg. The chemical composition of this extract identified 31 phenolic compounds, which includes catalagin, and ellagic and gallic acids [203].

*Salvia miltiorrhiza* Bge. (*S. miltiorrhiza*) stems’ and leaves’ total phenolic acids extract (JF) and *S. miltiorrhiza* roots and rhizome tanshinone extract (DT) have been investigated. Peng and colleagues studied if these extracts had a good anti-inflammatory effect and the potential molecular mechanisms of these extracts studied alone or in combination using the mice model with colitis induced by dextran sulfate sodium (DSS). Colitis was induced with 2% DSS in drinking water during 7 serial days, and then the mice were administered *po* for 7 days with DT and JF either alone or in combination. Researchers concluded that the combination of DT and JF had a superior inhibitory effect on inflammatory factors as compared to JF alone. It was also found that DT alone and JF combined with DT demonstrated an effective anti-inflammatory effect by inhibiting TLR4/PI3K/AKT/mTOR signaling-related proteins expression levels (including TLR4, NF-κB p65, p-AKT (ser473)/AKT, p-PI3K p110α/PI3K p110α, mTOR, p-mTOR) [204]. A flavonoid called hesperidin methyl chalcone (HMC) has been utilized to care for chronic venous disease, which demonstrates antioxidant, analgesic, and anti-inflammatory features in pre-clinical studies. HMCs effects on colitis, however, have never been studied. Guazelli and colleagues described the protective characteristics of HMC in a mouse model of colitis induced by acetic acid. Treating the mice with HMC substantially reduced neutrophil infiltration, colon shortening, edema, micro and macroscopic damages induced by the intracolonic acetic acid administration. After treatment with HMC, colitis improvement was associated with the rise in colon antioxidant status and the inhibition of pro-inflammatory cytokines IL-1β, IL-6, IL-33, and TNF-α in the colon. It was observed that HMC also inhibited NF-κB activation in the colon, which could explain the decrease in cytokines the research group observed [205].

A study with dextran sulphate sodium (DSS)-induced murine colitis by targeting the inflammasome NLRP3, demonstrated that genistein inhibited NLRP3 inflammasome through macrophage TGR5-cAMP signaling, demonstrating that this could be a potentially effective drug for IBDs. It was also shown that the manufacture of caspase-1 and IL-1β was down-regulated and augmented intracellular cAMP level, the same effect identified in vitro with semi-synthetic TGR5 agonist [206].

Several authors have already demonstrated (making use of the same in vivo model) that dietary genistein alleviates DSS-caused colonic injury by reducing colonic weight, rectal bleeding, and diarrhea ratio. Genistein downregulated the expression of cytokines, improved colonic permeability and barrier in DSS model resulting in a reduction in colon inflammation. In the in vitro model with Caco-2 cells, these authors proved that genistein enhanced cellular permeability and cell viability and repressed DSS-induced triggering of TLR4/NF-κB signal. They concluded that genistein eased colonic injury caused by DSS, gut dysfunction, and inflammation, which may be associated with the signal TLR4/NF-κB [207].

Genistein oral administration in TNBS-induced chronic colitis rat model was also investigated and it exerted advantageous anti-inflammatory effects in this rodent model via colon expression of COX-2 mRNA and protein reduction and decrease in myeloperoxidase activity [208].

Resveratrol (3, 4, 5-trihydroxy-trans-stilbene), present in various common foods such as grapes, berries, and peanuts, is a potent anti-inflammatory and antioxidant agent that activates the NAD-dependent deacetylase sirtuin 1 (Sirt1), which downregulates and inhibits the transcription activity of adipogenic factors such as PPARγ and C/EBPα [64]. Research shows that gut microbiota modulation through resveratrol supplementation has demonstrated a potential approach as a therapy for IBD [209].

Resveratrol (as well as its derivatives) has demonstrated potential as a therapy for the treatment and prevention of diverse chronic diseases such as IBD and diabetes [210,211,212,213]. Poor oral bioavailability is a result of the low solubility of resveratrol in water which limits the concentration in the plasma of resveratrol [214,215]. Absorption of resveratrol cannot be made in its native form; however, at a very low concentration in plasma, it exists in conjugated forms such as glucuronide and sulfate conjugates [216,217,218]. After oral administration, given resveratrol’s poor absorption, it has been reported that an accumulation in the large intestine can take place [219,220].

The intraperitoneal administration of resveratrol for 5 days before inducing colitis at a 10 mg/kg/day concentration considerably lowered malondialdehyde (MDA) and microscopy score levels and elevated glutathione peroxidase (GSH Px) activity as compared to TNBS and vehicle groups. A minor increase in catalase (CAT) activity was also witnessed in the group treated with resveratrol compared to TNBS and vehicle groups [211].

To guard resveratrol from rapid degradation, intensify its intestinal permeation, and alter its pharmacokinetics, PLGA nanoparticles loaded with resveratrol targeted with folate were created. Therapeutic efficacy of this system was then assessed in the subduing of colon inflammation on TNBS-induced colitis model. Resveratrol encapsulation into biodegradable folate targeted PLGA nanoparticles could present a powerful mechanism in hindering colonic inflammation, demonstrating a great potential for clinical translation [221].

The work of Sun et al. applied in a radiation-induced inflammatory bowel disease showed that resveratrol acts against the development of this inflammation through the inhibition of IL-1β expression, because resveratrol induces a Sirt1 level rise, which additionally suppresses NLRP3 inflammasome expression and consequent IL-1β production and secretion [222].

The team composed by Wang and his colleagues investigated the expression of a small ubiquitin-like modifier protein 1 (SUMO1) and its pathway-related genes Wnt/β-catenin. Results showed that treatment with resveratrol considerably alleviated colitis symptoms in a mouse model induced by DSS. Anti-inflammatory cytokines expression levels were augmented while those of pro-inflammatory cytokines were reduced in both spleen and colon tissues of mice treated with resveratrol. SUMO1 expression and the Wnt/β-catenin pathway was curbed in spleen and colon tissues of resveratrol-treated mice with colitis. Resveratrol repressed the expression of β-catenin and SUMO1 and their nuclear localization in the human colonic epithelial cell line (FHC). Higher expression levels of SUMO1 and β-catenin were found in patients with colorectal cancer patients than in healthy and colitis patients. Resveratrol alleviates DSS-induced colitis by controlling SUMO1 through the Wnt/β-catenin pathway [223].

A randomized, double-blind, placebo-controlled pilot study was performed in order to evaluate the resveratrol supplementation and oxidative or anti-oxidative grade in patients with UC. This study revealed that supplementation of 500 mg/day of resveratrol throughout 6 weeks can mitigate the disease activity and improve quality of life in these patients, partly at least by diminishing oxidative stress. Serum level of SOD and TAC augmented, whereas serum malondialdehyde reduced significantly in resveratrol group [224]. This supplementation also led to a significant reduction in plasma levels of TNF-α, PCR and the activity of NF-κB in peripheral blood mononuclear cells [225]. Resveratrol supplementation significantly reduced disease activity and improved the quality of life, assessed by the Simple Clinical Colitis Activity Index Questionnaire and Inflammatory Bowel Disease Questionnaire-9 [224]. It remains to be seen if these effects will keep for longer treatment periods. Further studies are, however, needed in order to determine ideal supplementation dosage for these patients [224,225].

An effective inhibition of colon cancer cell invasion and proliferation was also noted in in vitro studies with HT-29 cells; using these phenolic extracts, such as Mediterranean aromatic plants, might be a useful pharmacological tool for the adjuvant management of IBD, with a potential role in the supplementary therapy of IBD patients, and which may open up new research opportunities in the impairment of colon cancer progression. So much so, given the important link between inflammation and cancer, and the role of inflammatory processes in the progression of colorectal cancer [198,199].

Colitis-associated colorectal cancer (CAC) is a tumor that matures in the context of chronic inflammation and is seen as the most severe complication of IBD [226]. Additionally, CAC has a higher malignant potential than sporadic colorectal cancer (CRC), and the characteristically advanced stage of CAC in diagnosis lowers life expectancy [227]; CRC is estimated to account for 10–15% of IBD-related deaths [228].

The use of foods with anticancer and antimetastatic activity has opened doors for the nutraceutical prevention of tumor formation, in which foods or their components provide benefits for the prevention and/or treatment of tumors [229]. Certain successful therapeutic strategies against colon cancer growth have been attained through the combination of dietary and nutraceutical supplements with plant food matrix metalloproteinase inhibitors (MMPIs) for prevention, treatment, or postoperative relapses [230].

#### 2.1.3. The Microbiota Link between Rheumatoid Pathology and Inflammatory Bowel Disease

Clinical observations have shown the importance of intestinal involvement in systemic rheumatic pathology. Changes in host microbiota in the gut may therefore contribute to the pathogenesis of inflammatory disease in distant joint tissues [231,232]. The terminal part of the ilium produces inflammatory cytokines, including IL-1 and IL-23, in the presence of commensal microbes [233], and their excessive local production is associated with immune pathologies leading to IBD and the release of these cytokines into the bloodstream may promote rheumatic disease in distant locations. Human ankylosis and spondylitis are accompanied by IL-23 overproduction in the terminal ilium [234]. In fact, according to the results of Ciccia et al. (2009), overexpression of IL-23, but not IL-17, is a fundamental feature of subclinical bowel inflammation in ankylosing spondylitis. The identification of resident Paneth cells as an essential source of IL-23 under physiological and pathological conditions strongly suggested that IL-23 is a master regulator of intestinal mucosal immunity, providing pathophysiological significance for the reported association between receptor polymorphisms IL-23 and intestinal inflammation [234].

## 3. Phenolic Compounds

### 3.1. Importance for Human Health

Phenolic compounds are found in a wide variety of foods available for the human diet, including fruits, vegetables, beverages, herbs and spices, many of which have been used empirically by humans for thousands of years, including traditional medicine [52]. Several classes of phenols have been categorized on the basis of their skeleton as attested by Table 1.

In 1999, King et al. selected the three most essential groups of dietary phenols: polyphenols, phenolic acids, and flavonoids [238]. Several classes of phenols can be differentiated according to number of phenol rings and to the structural elements that join these rings [239]. Flavonoids are the largest and the most studied group of plant phenols.

A wide variety of biological activities attributed to flavonoids has been reported over the years such as: antioxidant activity; anti-inflammatory; hepatoprotective effect; antibacterial activity; antiviral; anticancer and antidiabetic activity [240,241,242,243]. The antioxidant properties of these molecules were explained based on the availability of the -OH groups and conjugated double bond system present in these molecules [85].

Dietary flavonoids have the capacity to mitigate inflammation by pursuing different intracellular signaling pathways activated by AP-1, MAPKs, NF-κB, PPAR and nuclear redox factor (Nrf2) [244].

*In vivo* studies in animal models have shown inhibition of some of the inflammation mechanisms as well as effects on the mechanisms of tumorigenesis by several of these compounds [52]. Even the consumption of small amounts of phenolic compounds has had a potent effect on reducing chronic inflammatory diseases in animal models, as well as in populations consuming foods rich in certain phenolic compounds as evidenced by the epidemiological studies [16,52,245].

Numerous studies between 2000 and 2016, from clinical trials to experimental research, reported on the anti-inflammatory potential of flavonoids, ascribing their ability to reduce inflammation not only to antioxidant effects, but also for the capacity to modulate various signaling pathways, such as JAK-STATs, NF-κB, and MAPKs [244].

Phenolic compounds mediate cellular signaling modulation and are therefore able to influence various cellular processes such as signaling, proliferation, apoptosis, redox balance, differentiation, etc. [85]. Additionally, they model NF-κB activation, NrF2 activation, glutathione biosynthesis, chromatin structure, ROS capture directly or via glutathione peroxidase activity, and, as a consequence, regulate genes involved in inflammation in macrophages and pulmonary epithelial cells. However, data propose that dietary phenolic compounds may function as changers of signal transduction pathways to exert their valuable biological effects [85].

Dietary flavonoids were studied between 2011 and 2015 for their anti-inflammatory potential and their effects on intracellular signaling pathways and inflammation and were accounted in at least 126 studies [244]. In these, dietary flavonoids that modulate the signaling pathways related with inflammation regulate the expression of pro-inflammatory mediators. The relationship between the structure of flavonoids and their anti-inflammatory activity is also evidenced, hoping to offer useful information for the development of new sources of natural-based anti-inflammatory drugs [244].

Simultaneously, pro-oxidant activity is one of the systems of action that flavonoids have against protozoan parasites [246,247]. Oxidative stress in the parasite can be escalated by flavonoids by accepting electrons from oxidoreductases that are unique to the parasite, that act as pro-oxidants in this case [247,248]. In addition, it could alleviate the secondary oxidative stress generated by defense cells against the infectious agent, which is likewise very toxic to nearby host tissues [249,250,251].

Some characteristics for the effective pro-oxidant activity of flavonoids have been well established. The pro-oxidant and antioxidant characteristics of flavonoids rely on their environment and chemical configuration, directly proportional to the total number of hydroxyl groups and their concentration [252,253]. Pro-oxidant activity may be important in vivo if free transition metal ions take part in oxidation processes and may be significant for certain metal excess diseases [252].

Phenolic compounds and flavonoids can be understood as future pharmacological agents that can be used as antioxidants and anti-inflammatory agents to combat oxidative states [85,128,133].

Such studies are carried out nowadays with all kinds of plant sources, for example the *Epilobium* species or “Yaki Otu”, as it is generally known in Turkey, which means “plaster herb”. Young shoots of *Epilobium stevenii Boiss., Epilobium hirsutum* L., and *Epilobium angustifolium* L. are typically consumed as part of a meal or as a salad. In traditional medicine, these species have been utilized as a dressing for treating mouth wounds, and a balm prepared from leaves has been used for children’s skin disorders. In vivo and in vitro experimental models were used to classify the active wound-healing component(s) and to describe the possible underpinnings of the wound-healing action. The active ethyl acetate (EtOAc) sub-extract of the aerial part of *Epilobium angustifolium* presented notable wound healing activity with antioxidant, anti-hyaluronidase and anti-collagenase action. Hyperoside (quercetin-3-*O*-galactoside) was classified as the principal active component of the aerial parts. According to these data, it was suggested that EtOAc sub-extract of *E. angustifolium* and hyperoside may be powerful candidates for the advancement of a wound-healing agent [254]. Antioxidant and anti-inflammatory activities are still being verified for hyperoside to this day [255,256,257].

Other examples of plant food, important to human diet, have been investigated in order to establish the health potential of their bioactive phenolic compounds’ ingestion.

Several species of buckwheat are cultivated worldwide, however, common buckwheat (*Fagopyrum esculentum*) and tartary buckwheat (*Fagopyrum tataricum*) are among the most extensively cultivated for use as human food crops [258]. Pseudo-cereals have received increased interest in recent years due to the growing awareness for the need for more healthy diets. Tartary buckwheat (*Fagopyrum tataricum Gaertn.*) is a pseudo-cereal rich in dietary beneficial components [259]. Compared to other cereal crops, buckwheat has heightened antioxidant activity, an aspect that has been ascribed to its high content of flavonoid compounds [260]. The inflorescences of these buckwheat variants (*Fagopyrum tataricum rotundatum, Fagopyrum esculentum*, *F. esculentum*, and forma green-flowers) were analyzed comparatively on total phenolics, phenolic acid composition and antioxidant activities. Using HPLC, eight phenolic acids (chlorogenic acid, ferulic acid, trans-ferulic acid, salicylic acid, p-coumaric acid, vanilic acid, p-anisic acid, and methoxycinnamic acid) were recognized. Inflorescences of *F. esculentum*, green flowers, contain an elevated degree of *p*-anisic acid [(872 mg/100 g dry weight (DW)] and chlorogenic acid (16 mg/100 g DW). The highest amount amongst the investigated buckwheat inflorescences of p-anisic acid, vanillic acid, chlorogenic acid, and trans-ferulic acid was found in *F. tataricum,* while *F. esculentum* inflorescences have been described as having the highest content of methoxycinnamic acid (74 mg/100 g DW) and salicylic acid (115 mg/100 g DW) [261].

Necessarily, more studies are necessary for a better understanding of the action of ROS on cellular functions in different cell types and the pathological impact of different inflammatory disease states [85], as efforts to recognize new and more effective antioxidants to be used in therapeutic strategies should carry on [85,133].

### 3.2. Phenolic Compounds and the Gut Microbiota Modulation

According to the statement issued by the International Scientific Association for Probiotics and Prebiotics, the description of a prebiotic has been freshly changed to “a substrate that is selectively utilized by host microorganisms providing a health benefit” [262], including the impact of prebiotic at extra-intestinal sites: on bone strength, on neural and cognitive processes, on immune functioning, skin, and on serum lipid profile [262,263]. Additionally, the term synbiotics describes both probiotics and prebiotics combined synergically, so this term should be restricted for products where the prebiotic compound(s) selectively favor the probiotic organism(s) [264].

Intestinal microbiota catabolizes phenolic compounds into smaller molecules that are better absorbed and may have beneficial health biological effects at the gut level, or elsewhere, circulating in plasma. In turn, phenolic compounds modulate microbiota by promoting the “*prebiotic-like effect*”, with the growth of beneficial microorganisms such as *Akkermansia* spp. and *Faecalibacterium* spp., and decreasing the *Firmicutes*/*Bacteriodetes* ratio, which is considered an advantageous effect. Differences in the intestinal microbiota population determine the metabolism of phenolic compounds, producing different metabolites, i.e., different metabotypes can modulate different health effects. In fact, some metabolites are more abundant in diseases or disorders with intestinal flora dysbiosis [265].

Dysbiosis is described as aberrant microflora, which probably has repercussions for immune function given that the exhaustion of commensal species is connected to the depletion of immune cell populations crucial for coordinating immune responses [266].

Cases of diseases concomitant with microbial dysbiosis include autoimmune and allergic diseases [267,268], IBD, obesity [269], diabetes [270], metabolic syndrome, and colorectal cancer [265].

*Bacteroides vulgatus* and *Prevotella copri* were recognized as the leading species motivating the link between insulin resistance and biosynthesis of branched-chain amino acids, and in mice it was demonstrated that *P. copri* can promote insulin resistance, exacerbate intolerance to glucose and increase flowing levels of branched-chain amino acids [271].

Bacterial species that deglycosylate food phenolic compounds in the gut include *Bacteroides*, *Enterococcus*, *Bifidobacterium*, *Blautia*, *Eubacterium* and *Lactobacillus* [272]. The microbiota that cleaves the polyphenol nucleus and reduce double bonds, dehydroxyl and demethyl mainly belong to the families *Coriobacteriaceae*, including species of *Adler-creutzia*, *Eggerthella*, *Gordonibacter*, *Paraeggerthella*, *Slackia*, and *Clostridiaceae* (*Clostridium* and *Flavonifractor*). The *Coriobacteriaceae* family is particularly interesting because it has been connected to advantageous metabolic effects on obesity and diabetes [273].

Obesity, closely tied to the microbiota, is a physiological state that has grown into one of the primary health concerns for populations that have adopted a westernized diet [274]. Within animal models of obesity, the interaction between the dominant gut phyla (*Firmicutes* and *Bacteroidetes*) is altered with a substantial subduing of the former and a corresponding rise in the latter [275]. This same trend has also been witnessed within individual humans on weight-reduction diets [274].

An increase in *Bifidobacterium* spp. has been shown to also modulate inflammation in obese mice by augmenting the manufacture of glucagon-like peptide-2, which has a reductive effect on intestinal permeability and, as such, decreases the translocation of lipopolysaccharides [276,277].

The significance of the interaction between the immune system and microbiota in obesity was shown in a study with genetically modified mice that lacked TLR5, which identifies flagellin and is one of the main microbial receptors of the innate immune system [278]. These mice developed attributes of metabolic syndrome altogether with substantial variations in their gut microbiota. It is conjectured that alterations in gut flora induce a low-grade inflammatory signaling that ultimately results in the development of metabolic syndrome. In addition, this obesity phenotype is communicable to wild-type mice just by transferring the microbiota [278].

Phenolic compounds could have an effect on gut microbiota composition [279,280,281,282].

The anthocyanins considerably instigate the growth of *Enterococcus* spp., *Bifidobacterium* spp., and *Lactobacillus* [280], suggesting that anthocyanins will, possibly, positively select for beneficial participants of the gut microbial community [280].

The modulatory impact on human intestinal microbiota by purple sweet potato anthocyanins (PSPAs) demonstrated that PSPAs prompted the spread of *Bifidobacterium* and *Lactobacillus*/*Enterococcus* spp., subdued the development of *Clostridium histolyticum* and *Bacteroides-Prevotella*, and did have an impact on the total number of bacteria. Greater influence on intestinal microecology may be exercised by PSPAs that were partly fragmented to phenolic acids during fermentation. This suggests that PSPAs may have prebiotic-like activity by producing short-chain fatty acids and modulating the intestinal microbiota, which contributes to improvements in human health, such as cardio-protective effects, antioxidant capacity, anti-inflammatory properties, reduction in diabetes risk, and inhibition of tumor cell growth, especially those in the colon [280,283,284,285,286].

Resveratrol has also been described as being able to modulate gut microbial composition while microbiota can adjust the biotransformation of resveratrol as well [279,287]. In resveratrol-fed mice, *Enterococcus faecalis*, which is associated to elevated levels of extracellular O^2−^, was considerably reduced. Likewise, research indicates that the relative abundance of *Bifidobacterium, Bacteroides*, *Lactobacillus*, and *Akkermansia* is amplified with resveratrol supplementation [288]. Prior studies showed that resveratrol administration can lead to surges in the number of *Lactobacilli* and in lowering the number of *Escherichia coli* and *Enterococcus faecalis* species in mice fed with a high-fat diet. It was also demonstrated through cellular studies that the antimicrobial action of natural phenolic compounds such as kaempferol and resveratrol, is a development blocker of *Enterococcus faecalis* [289].

The DSS-colitis rat model has shown that treating with 1 mg/kg/day of resveratrol for 25 days offers advantageous effects for the colon, which includes defending the architecture of the colonic mucosa, modifying the manifestation of inflammation-associated genes, and modulating intracellular signaling such as the NF-*κ*B signaling pathway [290]. Additionally, the administration of resveratrol can reestablish regular intestinal microbiota in bacterial composition by treating with DSS, which include some anti-inflammatory gut microbiota such as *Bifidobacteria* and *Lactobacilli* [291].

When mango pulp (*Mangifera indica* L.) was given as an adjuvant treatment combined with conventional medicine in patients (10 volunteers with mild/moderate IBD received for 8 weeks a daily dose of 200 to 400 g of mango pulp), mango intake altered beneficially fecal microbial composition by increasing significantly the abundance of *Lactobacillus reuteri*, *Lactobacillus* spp., *Lactobacillus lactis,* and *Lactobacillus plantarum*, followed up by an increase in the production of fecal butyric acid. Enriching a diet with mango fruits or other gallotannin-rich foods, therefore, seems to be an auspicious adjuvant therapy, especially when combined with conventional prescriptions in the management of inflammatory bowel disease by decreasing biomarkers of inflammation and modulating intestinal microbiota [200].

The manipulation of the microbiota as a therapeutic option is showing some promise for irritable bowel syndrome (IBS), a functional gastrointestinal disorder that is chronic and stressrelated. *Cynanchum thesioides* (CT) is an herb used in traditional medicine in Mongolia that has been used for hundreds of years in dealing with abdominal pain and diarrhea. Phytochemical studies of this plant exhibited the existence of numerous flavonoids with anti-inflammatory and antibacterial activities. Rat models of visceral hypersensitivity by maternal separation (MS) were tested for 10 consecutive days with CT water extract. This CT water extract acted beneficially against IBS visceral hypersensitivity and affected in favorable manner the functionality, composition, and structure of gut microbiota. After treatment with CT, gut microbiota stability was enhanced, and it was verified that the genera *Lachnospiracea incertae sedis*, *Pseudomonas*, and *Clostridium XlVa* (which were more frequent in MS rats) were considerably curtailed. The abundance of some genera, however, was less predominant in MS rats, which was the case, for example with *Clostridium sensu stricto*, *Acetatifactor, Clostridium IV*, and *Elusimicrobium*—all of which were expressively enriched after treatment with CT [292].

Zuojin Pill (ZJP) is a traditional Chinese medicine prescription composed of *Coptis chinensis* and *Evodia rutae-carpa* in a ratio of 6:1. ZJP has long been used to treat gastrointestinal diseases and has demonstrated positive therapeutic effects on experimental colitis [293,294]. Luo and colleagues reported that ZJP increases the proportion of CD4+ and CD8+ cells and decreases the concentration of proinflammatory cytokines tumor necrosis factor (TNF)-α and interleukin (IL)-1β [294]. However, the detailed mechanism of action of ZJP has not been defined. In order to achieve this, Zou and colleagues tested mice with DSS-induced colitis and treated them with ZJP for 7 days. ZJP’s therapeutic effect was assessed through microscopic and macroscopic observations, gut microbiota composition was established by 16S rRNA analysis, regulatory T (Treg) cells and their subsets were studied using flow cytometry technology, and the activation of the phosphoinositide 3-kinase (PI3K)/Akt signaling pathway was tested by Western blotting. These authors verified by pathological observation that ZJP decreased mucosal necrosis and inflammatory cell infiltration compared to the DSS group without treatment, resulting in a lower histological colitis score. The treated groups had significantly decreased expression of pro-inflammatory cytokines content in the colonic mucosa: IL-2, IL-6, IL-17A and IL-4. When stimulated by antigen, both PD-1 and PD-L1 were highly expressed on CD4+CD25+Treg cells. However, the percentage of PD-1+/PD-L1+Treg cells was reduced by ZJP. The data presented in this work revealed that ZJP inhibited expression of PD-1 and PD-L1 on the surface of CD4+CD25+T cells in colitis mice. Mice from the normal group showed a higher abundance of *Actinobacteria, Corynebacteriales and Micrococcales,* while mice from the DSS group showed a higher abundance of bacterial genera *Blautia* and *Aloprevotella* as well as *Lachnospiraceae*_NK4A136_group. At the phylum level, *Firmi-cutes* decreased the most significantly in the DSS group mice treated by ZJP. At the class and genus level, the relative abundance of *Verrucomicrobiae* and *Akkermansia* was noticeably increased after ZJP administration. Significant enrichment of *Verrucomicrobiaceae* and *Akkermansia* in the DSS +ZJP group was also found, and additionally, it was noticed that ZJP markedly decreased the levels of *Lachnospiraceae*_NK4A136_group compared to the DSS group without ZJP administration [295].

The impact of long-term feeding with phenolic compound (PC)-rich grape pomace extracts on the rat microbiota was tested using different concentrations of PC (20, 10, 5 and 2.5 mg/kg/day). Major phenolic compound components were characterized by HPLC, and DPPH assay was used to measure free radical scavenging capacity. Fecal samples taken from young, 2-month-old rats, and rats fed every day with DMSO or PC were collected at 6- and 14-months post-treatment. In order to analyze gut microbiota, composition of q-PCR was applied. The growth of probiotic bacteria such as *Bifidobacterium* was significantly higher in the groups, PC 2.5 and PC 5, as compared to the control group and the young rats group. In all treated and untreated groups, *Lactobacillus* levels diminished with time. *Enterococcus*, *Clostridium leptum* subgroup (*Clostridium* cluster IV), and *Bacteroides* were not substantially changed by phenolic compounds at any dose when compared to control. However, after being treated for 14 months, all phenolic compound doses eliminated the rise of *Clostridium sensu stricto* (cluster I) as witnessed in the control group in comparison to young rats. Gut microbiome in rats is selectively modulated by phenolic compounds to a healthier phenotype in long-term feeding rats and might counter the detrimental outcomes of aging on the population of gut bacteria [296].

Given that phenolic compounds might exert prebiotic activity, it is critical to comprehend their stimulatory or inhibitory effects on pathogenic or advantageous bacteria [297].

Given the impacts in health as a result of their antiestrogenic, cardioprotective, antioxidant, anti-inflammatory, neuroprotective and cancer chemopreventive properties, a current trend in human nutrition and in phenolic compounds research is the classification of PC’s produced metabolites by gut microbiota [298]. As examples, we have equol, which is transformed from soy isoflavones, urolithins, which are transformed from ellagitannins, dihydroresveratrol, which are transformed from resveratrol, 8-prenylnaringenincereal, which is transformed from hop (beer) isoxanthohumol, and mammalian-lignans (enterolactone, enterodiol), which are transformed from lignans [299]. The hydrolysis of chlorogenic acid by *Bifidobacterium animalis subsp. Lactis*, as well as the transformations of dihydroresveratrol from resveratrol by *A. equolifaciens* and *S. equolifaciens*, has also been reported in the last years [300,301].

Ellagitannins can be found in the following: tea, pomegranates, walnuts and other nuts, muscadine grapes, oak-aged wines, and berries such as arctic bramble, blackberries, cloudberries, raspberries and strawberries [299,302]. In order to produce ellagic acid (EA), lactonization of ellagitannins occurs after undergoing hydrolysis in the gut. Generally, it is accepted that ellagitannins and EA intestinal absorption is very low and when they reach the colon, they are catabolized by the gut microbiota into urolithins—these metabolites being much more absorbed [303,304,305]. Due to the urolithin effect in health, the recognition of bacteria responsible for urolithin production is a relevant outcome (anticarcinogenic, anti-inflammatory and cardiovascular protection properties). Bacteria production of urolithin could be applied as probiotics or in industrial production to develop drinks, food ingredients, dietary complements, pharmaceuticals, and functional foods enriched in urolithins produced in a similar way as it is produced in the intestine [306]. Urolithins are metabolites that are bioavailable and can reach up to micromolar concentrations in human plasma [304]. Poor bioavailability of EA or ellagitannins, as well as the biological effects of urolithins in different in vitro assays, suggests that urolithins can be the bioactive molecules in vivo responsible for the health effects observed after ellagitannin or EA intake [307]. These metabolites exert anti-inflammatory [308,309,310,311,312,313] and cancer chemopreventive effects against colon cancer [313,314].

In addition, intestinal microbiota has an impact on the biotransformation of phenolic compounds [297]. Nevertheless, reciprocal interactions have been demonstrated [279,280,281,282,297], yet much remains to be understood.

### 3.3. Phenolic Compounds Bioavailability

The beneficial effects of phenolic compounds depend on the amount consumed but also on their bioavailability: the study of absorption, distribution, metabolization and elimination mechanisms (ADME) is essential for a better building of experimental studies and for understanding the observed effects [52,315].

The bioavailability of dietary compounds, in general, and phytochemicals, in particular, depends upon their release from the food matrix, their digestive stability and the competence of transepithelial passage. The best way to determine the benefits of food intake and bioavailability is to subject the product to gastrointestinal digestion in vivo, despite all ethical, medical, financial and long observation periods, especially when referring to the product to humans [316].

Absorption and metabolism of food constituents that have phenolic groups are determined by chemical structure, hence the degree of glycosylation/acylation, the basic structure present, conjugation with other phenolic groups, molecular size, degree of polymerization and solubility [317]. The chemical structure of phenolic compounds further governs the rate and degree of intestinal absorption and the nature of circulating metabolites in plasma [318]. Due to hydrophilicity, glycosylated flavonoids pierce the cell membrane with effort [244]. The flavonoid aglycone and glycosylated flavonoids are absorbed in the small intestine, but are rapidly transformed into methylated, glucuronidated or sulforated derivatives [319]. Some bioavailability studies in humans have shown that the amounts of phenolic compounds found intact in urine vary from phenolic compound to phenolic compound [318].

Upon consumption, flavonoids in food experience considerable biotransformation within the small intestine and within the liver (methylation, conjugation, oxidation, deglucosylation), but in the large intestine (double bond disruption, ring cleavage, decarboxylation) as well. Consequently, after ingestion, traces of the flavonoid can be identified in plasma; predominant in systemic circulation and tissues are flavonoid metabolites. Flavonoid metabolites have a lower antioxidant strength than ingested molecules and, in addition, some of them (catechol free fraction metabolites and/or free hydroxyl group at position C-3) showed pro-oxidant effects (increased production hydrogen peroxide radicals and superoxide anion). Pro-oxidant activity, however, may be useful as it creates a rise in detoxifying enzymes and antioxidant systems of defense [320].

However, even with their health-promoting effects, the use of anthocyanins has been restricted by their low chemical stability under physiological conditions after oral consumption by humans [321]. As they pass to the intestine (pH changes from acidic to approximately neutral or mildly alkaline), anthocyanins are transformed to a blue quinoidal base by the loss of protons, which is an unstable form [322]. As a consequence, these transformations contribute to a low bioavailability of anthocyanins [323]. Encapsulation may provide a robust delivery mechanism that stabilizes anthocyanins and thus increases bioavailability in the intestine. Encapsulation with cyclodextrins is another example of this and it was demonstrated that it raised bioavailability of anthocyanins, permitting them to be distributed in the colon and to exercise their potential health benefits. A bacterial metabolization with liberated anthocyanins was registered and their metabolites might exert the positive modulation of the microbiota [324].

The oral bioavailability of quercetin and kaempferol in vivo was significantly enhanced by incorporating persimmon (*Diospyros kaki*) leaf extract in self-emulsifying delivery systems (where droplet size of dispersion ranges of 100–250 nm) when compared with commercial tablets. According to the correlation study in vitro–in vivo, the oral bioavailability augmentation was due to the rise of drug concentration in gastrointestinal tract and absorption area [325].

Nanoparticles resulting from edible ginger (GDNPs 2) were created and revealed their effective colon targeting when orally administered. The average size of GDNPs 2 was ~230 nm and they possessed a negative zeta potential. These nanoparticles are composed of great amounts of ginger bioactive compounds (6-gingerol and 6-shogaol), a few proteins, high levels of lipids, ~125 microRNAs and GDNPs 2 were mainly taken up by epithelial cells of intestine and macrophages and had no toxic effects. GDNPs 2, administered orally, increased the proliferation and survival of intestinal epithelial cells and reduced the pro-inflammatory cytokines (IL-6, IL-1β, and TNF-α), and increased the pro-healing anti-inflammatory cytokines (IL-22 and IL-10) in ulcerative colitis models, suggesting that GDNPs 2 reduced acute colitis, improved intestinal repair, and prevented CAC and chronic colitis [326].

The strategy for the controlled release of resveratrol was tested with silk fibroin nanoparticles (FNPs) (around 100 nm) loaded with resveratrol (resveratrol-loaded FNPs (RL-FNPs)) in the TNBS-induced colitis model. The tested groups were: unloaded FNPs; resveratrol; resveratrol encapsulated in FNPs (RL-FNPs); dexamethasone. All treatments were administered intrarectally.

Animal groups administered with fibroin nanoparticles carrying resveratrol exhibited a better impact than the other tested groups, with significant differences from the group of animals that received the unloaded nanoparticles; this effect was similar to that detected in the rat’s group that received dexamethasone. Animals with colitis administered with RL-FNPs expressively reduced the expression of TNF-α, IL-1β, IL-6, and IL-12, with effectiveness comparable to that of the dexamethasone group, with the exception of IL-12. The group administered with resveratrol had a considerable impact on TNF-α expression and unloaded FNPs expressively repressed the expression of IL-12 and IL-6. Dexamethasone did not stop the expression of CINC-1 and MCP-1, the two chemokines assessed. However, RL-FNPs’ administration was connected with a significant reduction of chemokines, CINC-1 and MCP-1, expression. Inhibition that was analogous to that achieved in the resveratrol group and the expression of MCP-1 was considerably reduce by unloaded FNPs [327].

Recently, in a work by Siu and colleagues, resveratrol-loaded galactosylated nanoparticles (around 108.4 nm in particle size) were studied for the oral bioavailability in rats and in vitro anti-inflammatory activity was investigated in lipopolysaccharides-induced RAW 264.7 cells. These nanoparticles significantly enhanced the oral bioavailability of RES; they effectively promoted the intestinal absorption of RES and strengthened its bioactivity, and the anti-inflammatory efficacy of these nanoparticles in the RAW 264.7 cell model was superior to free RES [328].

Limonin is a bitter compound that exists in some citrus fruits; it is a highly oxygenated triterpenoid that occurs as aglycones with a variety of biological activities, such as lowering cholesterol, anti-inflammatory activities, and acts as cytotoxic and cytostatic agents in animal and human cell cultures [329,330,331,332,333]. The suppression of the bitter taste of orange juice (phenomenon with a significant negative commercial impact) while keeping the healthy properties due to limonin was possible through the complexation of limonin in cyclodextrins (e.g., β and γ). This strategy is open to new industrial applications for cyclodextrins and health promotion [334].

The encapsulation of flavonoids in liposomal delivery systems in fact had demonstrated that the instability of dietary flavonoids can be improved. After being encapsulated, quercetin displayed the strongest lipid peroxidation inhibition capacity and scavenging of 2,2-diphenyl-1-picrylhydrazyl (DPPH), followed by kaempferol and luteolin [335].

Gallic acid phospholipid complexes, in different proportions, improves the lipophilic characteristics of gallic acid and overcomes its reduced absorption given its lower lipophilicity; these were developed by Shyam et al. [336]. Gallic acid is a constituent of traditional Chinese medicine and it is well known to have antioxidant properties and is commonly used as an additive in food, thus protecting health from oxidative stress damages, increasing survival [337,338]. The conjugation of gallic acid increases its bioavailability and augments its hepatoprotective effect in an experimental rat model where oxidative damage in liver is chemically induced [339].

Maiti et al. described that phytosomes of curcumin, a flavonoid from turmeric (*Curcuma longa*) displayed greater antioxidant activity than pure curcumin in all doses studied [340]. The use of supplements with phytosomal curcumin in subjects with NAFLD demonstrated that short-term curcumin supplementation improves liver fat and transaminase levels in patients with non-alcoholic fatty liver disease [341]. Curcumin combined with 5-Flurouracil in phytosomes used in a mouse-model of colorectal cancer subdued cell growth and the invasive nature of CRC cells through the modulation of the Wnt-pathway and E-cadherin. It also inhibited the colonic inflammation and significantly recovered the increased levels of MDA and decreased activity of CAT [342].

These new strategies of completely green nanotechnologies, such as phytosomes, can be employed to make the bioavailability of bioactive phenolic compounds more efficient [343], increasing the beneficial health effects of phenolic compounds that they carry, and may also be supportive of traditional pharmacological therapies in the context of disease [344,345].

The current changes in preferences, because of advanced knowledge surrounding the interplay between health and food, have caused a rise in new food supply demands. Nowadays, there is a growing interest in plant foods, exotic or not as well-known plant foods, not to mention underutilized or even discarded fruits. Plant foods are an important source of bioactive compounds with a positive health impact [45]. These new technologies of encapsulation can effectively revolutionize dietary habits, functional food production, and food supplementation. However, more studies are needed for a better knowledge of plants and their bioactive compounds, the efficacy in human health, the best delivery systems and production technology that can take them to food distribution shelves to the population.

## 4. Conclusions

Given the enormous human and economic toll of diseases related to unhealthy diets, longevity of modern populations, sedentary behaviors, pollution, etc., the development of measurable and reliable quality dietary habits and supplementation could be used for nutrition guidelines across various regions of the world.

Oxidative and inflammatory phenomena have an extremely important role in the pathophysiological processes of NCD etiology. Phenolic compounds are found across a wide diversity of foods available for the human diet. Phenolic compounds act as antioxidants, protecting human tissues against oxidative stress and conditions associated with this condition. These compounds can provide an excellent model for the development of more effective and, most importantly, safe future chemopreventive compounds. Food compounds have also a key effect on the gut microbiota, influencing its composition in terms of diversity and abundance. Dietary habits can intensely influence gut microbiota composition, which impacts the healthy state of individuals. A new nutritional approach may be adopted by constructing a personalized diet following the microbiota analyses, in order to modulate and repair a healthy gut microbiota. The new delivery systems within the field of nanotechnology could provide bigger efficiency of dietary compounds as phenolic compounds when ingested by humans, providing their health benefits in well-established ways of prevention, treatment or adjuvant in addition to conventional pharmacological treatments.

## Figures and Tables

**Table 1:** Classes of phenolic compounds categorized on the basis of their skeleton of C [235,236,237].

Skeleton	Classes
C_6_-C_1_	phenolic acid
C_6_-C_2_	acetophenone, phenylacetic acid
C_6_-C_3_	hydroxycinnamic acids, coumarins, phenylpropanes, chromones
C_6_-C_4_	naphthoquinones
C_6_-C_1_-C_6_	xanthones
C_6_-C_2_-C_6_	stilbenes, anthraquinones
C_6_-C_3_-C_6_	flavonoids, isoflavonoids
(C_6_-C_3_)_2_	lignans, neolignans
(C_6_-C_3_-C_6_)_2_	bioflavonoids
(C_6_-C_3_)_n_	lignins
(C_6_)_n_	catechol melanins
(C_6_-C_3_-C_6_)_n_	condensed tannins

## Data Availability

Not applicable.
